# Supplementary material for: Proteomic Changes in Paspalum fasciculatum Leaves Exposed to Cd Stress
Source: Plants (Basel). 2022 Sep 20;11(19):2455. doi: 10.3390/plants11192455 (PMC9573290; doi:10.3390/plants11192455)
Supplement: Supplementary file 1 [file plants-11-02455-s001.zip › Figure S1.pdf]

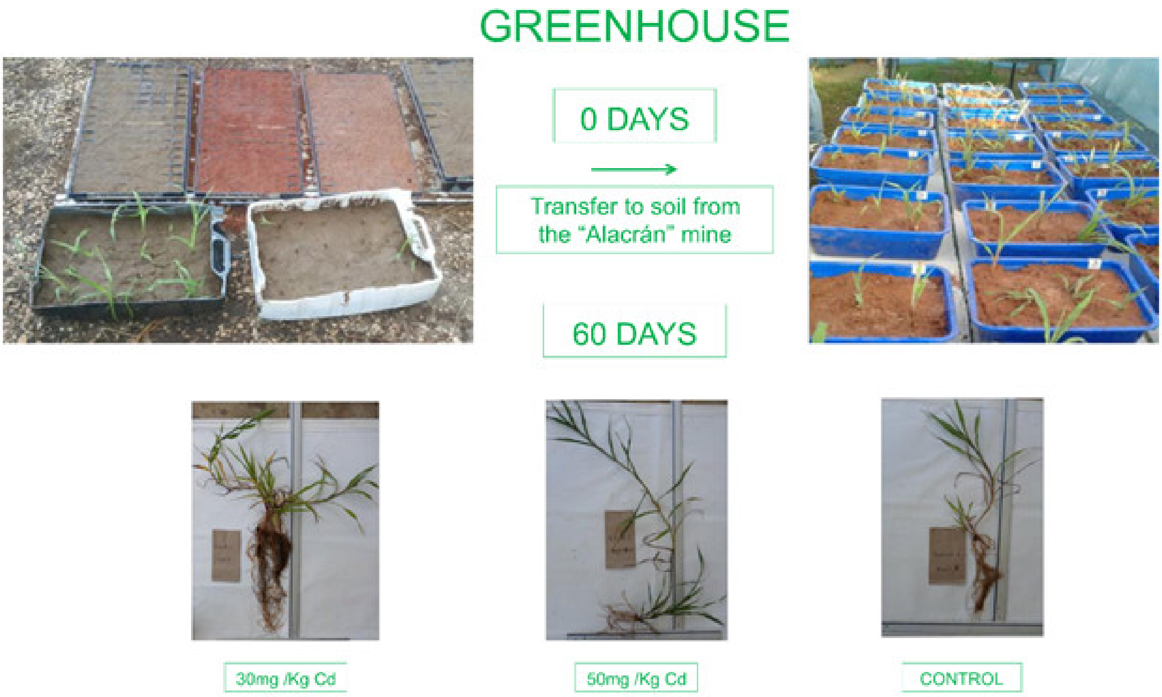

Figure S1. Reproduction by cuttings of *P. fasciculatum* plants in fertile soils and subsequent transplantation to mining soils supplemented with Cd during 60 days.
