# Supplementary material for: Proteomic Changes in Paspalum fasciculatum Leaves Exposed to Cd Stress
Source: Plants (Basel). 2022 Sep 20;11(19):2455. doi: 10.3390/plants11192455 (PMC9573290; doi:10.3390/plants11192455)
Supplement: Supplementary file 1 [file plants-11-02455-s001.zip › Table S2.pdf]

Table S2. Physical and chemical characteristics of the soil from “El Alacrán” gold mine (n = 3).

**Soil Properties**					
**Properties of Bioavailability**		**Texture**		**Metals(mg kg^{-1}) ± SD**	
**pH**	3.67 ± 0.03	Sand (%)	27.5 ± 0.03	Cd	7.27 ± 0.1
**OM (%)**	1.54 ± 0.09	Clay (%)	4.4 ± 0.09	Pb	2.72 ± 0.4
**CEC**	13.1 ± 0.01	Silt (%)	68.1 ± 0.06	Hg	0.5 ± 0.01
**Ca/Mg**	0.6 ± 0.02	-	-	Ni	25.2 ± 0.5
**Ca/K**	2.5 ± 0.3	-	-	Fe	13.1 ± 0.2
**Mg/K**	3.9 ± 0.03	-	-	Mn	19.1 ± 0.7

OM (%): organic matter percentage; CEC: cation exchange capacity; Sand (%): sand percentage; Clay (%): clay percentage; Silt (%): silt percentage.Ca/Mg, Ca/K, Mg/K: Cation ratios.
